# Still a moral dilemma: how Ethiopian professionals providing abortion come to terms with conflicting norms and demands

**DOI:** 10.1186/s12910-020-0458-7

**Published:** 2020-02-11

**Authors:** Demelash Bezabih Ewnetu, Viva Combs Thorsen, Jan Helge Solbakk, Morten Magelssen

**Affiliations:** 1 Department of Physiology, St Paul’s Hospital Millennium Medical College, Addis Ababa, Ethiopia; 2 Centre for Medical Ethics, Institute of Health and Society, University of Oslo, Pb. 1130 Blindern, N-0318 Oslo, Norway; 3 Department of Community Medicine and Global Health, Institute of Health and Society, University of Oslo, Oslo, Norway

**Keywords:** Abortion, Abortion politics, Moral status, Religious convictions, Moral values

## Abstract

**Background:**

The Ethiopian law on abortion was liberalized in 2005. However, as a strongly religious country, the new law has remained controversial from the outset. Many abortion providers have religious allegiances, which begs the question how to negotiate the conflicting demands of their jobs and their commitment to their patients on the one hand, and their religious convictions and moral values on the other.

**Method:**

A qualitative study based on in-depth interviews with 30 healthcare professionals involved in abortion services in either private/non-governmental clinics or in public hospitals in Addis Ababa, Ethiopia. Transcripts were analyzed using systematic text condensation, a qualitative analysis framework.

**Results:**

For the participants, religious norms and the view that the early fetus has a moral right to life count against providing abortion; while the interests and needs of the pregnant woman supports providing abortion services. The professionals weighed these value considerations differently and reached different conclusions. One group appears to have experienced genuine conflicts of conscience, while another group attempted to reconcile religious norms and values with their work, especially through framing provision of abortion as helping and preventing harm and suffering. The professionals handle this moral balancing act on their own. In general, participants working in the private sector reported less moral dilemma with abortion than did their colleagues from public hospitals.

**Conclusions:**

This study highlights the difficulties in reconciling tensions between religious convictions and moral norms and values, and professional duties. Such insights might inform guidelines and healthcare ethics education.

## Background

A central issue on the global public health and human rights agenda is abortion services. In many countries where the abortion law has been liberalized, abortion still gives rise to controversy both among health professionals and among the general public, not the least in countries where faith traditions and practices are prevalent, as is the case in sub-Saharan Africa.

### The path to liberalization of the abortion law in Ethiopia

The 1994 International Conference on Population and Development highlighted the need to prevent unsafe abortions and provide safe abortion services where lawful [1]. In the aftermath of the conference the liberalization of abortion laws in Africa has been promoted. African leaders agreed to address the problems constituted by unsafe abortion and lack of access to safe abortion through reforming national laws and policies, preparing service delivery guidelines and regulations, strengthening training programs, and expanding community outreach programs [2].

Throughout the 1990s, the abortion issue was put on the political agenda in Ethiopia. Advocates of liberalization wanted to reduce the incidence of unwanted pregnancies and save lives. Yet, they were met with opposition, often rooted in religious faith traditions and religious practices. In Ethiopia, the majority of the population regard themselves as religious: 44% are Orthodox Christians, 34% are Muslims and 19% are Protestants [3]. A 2007 study of the Ethiopian population showed that a majority (67%) regarded induced abortion as ‘never justifiable’ [4].

Relatedly, the Ethiopian population policy goal set in 1993 was to harmonize the rate of population growth with that of the economy. Among its many objectives were reduction of the high fertility rate from 7.7 to 4, and increasing the prevalence of modern contraceptive use among married women of reproductive age from less than 5% to at least 44% [5]. The principle that every pregnancy should be planned and wanted was incorporated into Ethiopia’s population policy.

In 2005, the Ethiopian abortion law was liberalized, making induced abortion legal after rape or incest, if the woman’s life or physical health is endangered, if she is physically or mentally disabled, or if she is a minor (less than 18). In addition, abortion is legal in the case of fetal impairment [6, 7].

### Abortion in Ethiopia

In Ethiopia, abortions are performed by several different healthcare professionals: nurses, midwives, health extension workers (community health workers with one and a half year of training), health officers, integrated emergency surgical officers, and doctors who are general practitioners or specialists or in training as gynecologists-obstetricians (GYN-OBS). The 2014 guidelines authorize integrated emergency surgical officers to give comprehensive abortion care for second trimester abortions [8]. From 2008 to 2014, the proportion of abortion-related services provided by non-physicians increased from 48% in to 83% [9]. Not much research exists about health professionals’ attitudes towards abortion; in one study, most practicing midwives were positive to provide abortion services and their attitude was positively associated with clinical experience [10].

Studies in 2008 and 2014 show that abortion services in Ethiopia have undergone rapid expansion and improvement since the introduction of the law in 2005, as assessed by the standards of the well-established ‘safe abortion care’ and ‘emergency obstetric care’ frameworks [11, 12]. An estimated 620,000 abortions were performed in 2014, corresponding to an annual rate of 28 per 1000 women aged 15–49. The proportion of abortions performed in health care facilities rose from 27% in 2008 to 53% in 2014. Two-thirds of abortions are performed in private/non-governmental organisation (NGO) centers (henceforth termed ‘private’, for ease of expression) [9].

Ethiopians’ knowledge of the abortion law is moderate. For instance, a survey of women aged 15–49 from Bahir Dar in North-Eastern Ethiopia revealed that two-thirds were aware of the existence of the new law, yet 57% had little knowledge of it [13].

### Research on abortion providers

Research on Ethiopian abortion practices has been sparse. In particular, the political, medical and ethical struggles over abortion among health professionals tasked with performing and assisting with abortion themselves have not been given much attention. A recent study from Addis Ababa, which parallels ours in involving interviews with abortion providers, describes health professionals’ struggle to balance religiously- and morally-based opposition to abortion against their professional duty to provide abortions and their concern for the women [14]. A key finding in this study was that religious anguish and the stigma associated with the job appeared to lead to burnout for some. Seeing as how the health care workers’ own attitudes towards the law and abortion practices varied, the researchers hypothesized that such attitudes would be likely to influence which patients would get access to abortion.

A national survey of physicians working in Ethiopian public hospitals showed that the respondents often experienced dilemmas related to reproductive health issues [15]. Respondents pointed to moral doubt and regrets in cases of abortion, as well as obligations to mitigate harm to women who might otherwise seek out unsafe abortions. Some respondents thought the abortion law was too strict, and that they were put in a dilemma when they found the abortion to be justified yet the woman did not fulfill the law’s criteria.

In a review of studies on Sub-Saharan and Southeast Asian healthcare professionals’ perceptions of and attitudes towards abortion, Loi and colleagues found that religion, among other factors, influenced attitudes towards abortion, and that professionals’ attitudes subsequently affected the relationship to the patient seeking abortion [16]. They noted that a majority of professionals support abortion after rape or incest to save the woman’s life, or when severe fetal abnormalities were discovered [16].

### Study setting and aims

Ethiopia’s total population is 105 million, of which about 3.4 million live in the capital Addis Ababa where the study took place. The capital is the most well-developed region in the country. Ethiopia spends on average $7.6 per capita on all dimensions of health care yearly. The fertility rate at 1.5 is half the national average [17]. The abortion rate is the highest in the country’s capital, estimated at 92 per 1000 women age 14 to 49. The 2014 national abortion rate 2014 was 17.6 per 100 live births [18].

As described above, in Ethiopia in the preceding three decades the international normative framework of reproductive human rights has clashed with traditional and religious opposition to abortion. In this study, we wanted to explore how health professionals experience and negotiate presumably conflicting allegiances. Thus, the aim was to shed light on health professionals’ moral reasoning and experience with regard to moral dilemmas surrounding abortion. In this article we report on the professionals’ views on the fetus and fetal moral status and how this is balanced with the right of the woman, and on the role and place of religion in professionals’ moral deliberations.

## Methods

### Design, study area and recruitment

Because the aim was to explore in depth the viewpoints and moral reasoning of healthcare practitioners assisting, performing or otherwise involved in abortion services, a qualitative study design was chosen. The intention was to recruit healthcare practitioners from a range of professional backgrounds involved in abortion services in either public hospitals or private abortion clinics in Addis Ababa.

The first contact was through the first author’s phone calls to the institutions. Each participant was given 100 to 200 Ethiopian Birr (approx. 3–6 Euro) for transport and compensation of time. Most of the 30 participants (22) provided abortions directly, while eight participants worked with abortion in an administrative capacity and/or provided contraceptives and post abortion care services. Half (15) of the participants were female. The participants had experience with abortion services ranging from 2 months up to 14 years.

### Interviews

Participants were interviewed at their workplace by the first author. Interviews were conducted between February and July 2017, and lasted on average 40 min. An interview guide was used, with open-ended questions on views on abortion, fetal moral status, the influence of religion on the participant’s views, and perceived moral dilemmas in abortion provision. All interviews were conducted in the Amharic language. Upon obtaining informed consent, interviews were recorded digitally. Then they were transcribed. The first author took field notes. An independent researcher translated the Amharic transcripts into English.

### Analysis

The transcripts were analysed by the first and last author using systematic text condensation (STC), a qualitative analysis framework developed by Malterud [19]. STC is a four-step model:

1. From chaos to themes: the transcripts and field notes were read several times to create an overall impression and identify candidates for main themes.
2. From themes to codes: each unit of meaning was identified and coded according to topic using the nVivo 11 software package. Codes and sub-codes were created.
3. From code to condensation: all units of meaning coded with the same sub-code were then read in order with a view to identifying their meaning and content. This was done by creating so-called ‘artificial quotations’, which are condensed summaries of salient points formulated as if phrased by the participants. All sub-codes were condensed in this way.
4. From condensation to analytic text: the artificial quotations then provided the basis for the final analytic text which was then incorporated into the Results section of the article. In the analytic text, genuine (not artificial) quotations from the transcripts are used to illustrate and confirm the findings.

## Results

In total, 30 health professionals (nurses, midwifes, public health specialists, general practitioners and OB-GYNs) were interviewed. Of these, 17 participants worked in public hospitals and 13 in private clinics. Participants presented a diversity of viewpoints on the moral status of the fetus and its implications with regard to abortion. Similarly, they exhibited different strategies to reconcile conflicting religious, ethical and professional duties. These viewpoints and strategies are presented below.

### The moral status of the fetus

We noted that when asked to give their view on when life begins, what moral value the fetus has and when it acquires a right to life, many of the participants hesitated and took time before answering. For some, for the fetus to have ‘life’ or to be considered ‘being alive’ was synonymous with being a ‘human being’ and having ‘human rights’ and a ‘right to life’. Some participants appeared to use the terms just mentioned both descriptively (e.g., biologically) and normatively (i.e., ethically and/or legally) at the same time. A majority, however, were clear in separating the descriptive and normative domains. Among the latter participants, a prevalent view was that life in a biological sense starts at conception. This was so even though they all went on to defend abortion in at least some situations as a matter of ethics and law, as exemplified by this participant:

Life begins at conception. I believe it has got a right from the beginning. … [Abortion] contradicts the fetus’ right to live. However, the mother also has to have a right, isn’t that right? Priority should be given to the mother. (#1, female nurse, private clinic)

There was a large variety of views on when the fetus acquires moral value and a right to life. Broadly, these fell into three main categories. First, there were those who stated that moral value and a right to life begin already at conception or implantation:

I say that [life] is from fertilization. Because, if it doesn’t have life, it doesn’t grow. [The embryo] is a proper person. That is the basis. … It has a right to live. It has a right from the moment it is conceived. (#7, female nurse, private clinic)

An unborn child has a right to life. God knows the fetus even before it is conceived. … I don’t have a right to abort it after conception. It has an owner. (#29, male OB-GYN, public hospital)

Second, some claimed that moral value and rights of the fetus come gradually throughout the development of pregnancy:

We do not believe that [the fetus] is a complete human being. … We do not look at it from a moral perspective. We look at it from the mother’s perspective. As [it is] a human being there are some feelings attached to it. However, we prioritize the mother. (#2, female nurse, private clinic)

Third, for others, moral value and a right to life begin either at birth, at viability, or at 28 weeks as per the Ethiopian abortion law:

I do not believe that a fetus just created has life. … When it is born and begins to breathe, I [say] that it has life. Especially after the 28th week. For me, after it is born … it has a right to live. (#5, male health officer, private clinic)

In general, more participants from private clinics held the second or third views than did those from public hospitals, who more often held the first view.

### Abortion in a religious perspective

When asked about the influence of religion on their abortion practices and viewpoints, several clearly had experienced a dilemma and a conflict of conscience. Most were clear that their religion had moral norms and values that condemn abortion more or less unequivocally.

For some, there was a genuine conflict between their practice and the religious condemnation of abortion, a conflict which continued to trouble their conscience. Some stated that they would also conceal the nature of their work from their acquaintances.

I am an Orthodox believer. I have a debate in my conscience, I have a guilty feeling. For me [abortion] is shortening life. I am not happy. … There are some who find it difficult. Everyone is doing it, although complaining. There are some who wonder [whether they should] change their field. (#19, female nurse, public hospital)

Previously I was not affected, but now as I am growing older, I sustain some feelings of guilt. Because this is definitely a sin. Nowadays I do not tell [people] that I work in [the abortion clinic]. (#9, female public health specialist, private clinic)

For others, the act of providing abortion services appealed to their religion’s moral norm of helping people in need, and they argued that this norm should have priority. They did not doubt that their religion in fact condemned abortion. However, they attempted to reconcile the conflicting moral norms and duties, whilst retaining a religious moral outlook.

Even though [abortion] stands in stark contrast with religion, at the same time people should not suffer. Therefore, when I do my job I reconcile the two. (#3, male public health specialist, private clinic)

Sometimes [abortion practices] get in conflict with religion. I reassure myself when I look at it from the angle of helping. I look at it from the angle of helping others, so, I do not believe that it is counted on me as sin. … God had said help those who are in need. (#5, male health officer, private clinic)

Several reflected on the experiences they had, especially with complications from unsafe abortions and on having changed their views in the course of their work.

When I started to see things and do them, I became more and more persuaded. I know, in religion, [abortion] is not permitted. I used to, from that perspective, think that all pregnancies must be born. I changed as time goes. [Whether] you are involved here or you see it from the outside, it is not the same. When you sit and hear people’s stories, your view changes gradually. (#17, female nurse, public hospital)

I am a Muslim but I am liberal. My view on abortion is liberal. The reason for that is that I had worked in the rural area. … I have seen 3–4 who lost their lives. … As long as they meet the requirements of the law, I have no reservation. … We need to save her life. Islam does not prohibit the termination of the pregnancy. … I have seen those who died because I rejected them. … I prefer to [perform abortion] because it is a matter of life and death. That is how my logic works. (#20, male GYN/OBS, public hospital)

For this group, performing abortions was justified by the serious needs it met in safeguarding the woman’s health. This also implied that only particularly weighty reasons for abortion (e.g., the woman’s health) would be sufficient to condone it:

I would like to do only reasonable and convincing abortion scenarios. I terminate early pregnancies, less than 5 weeks of gestation and incomplete abortions. Non-reasonable ones are not acceptable for me. (#30, male GYN/OBS, public hospital)

A final group stated that even though they were religious, their religion had little or no influence on their views and practice concerning abortion. This group had deliberately set religious norms and values aside and displayed no need to justify this further in the interview.

My thoughts are based on helping people in need of help, I do not bring it to my religion. (#10, female nurse, private clinic)

## Discussion

### Balancing allegiances and concerns

The aim of the study was to understand how professionals involved in abortion services experience and come to terms with presumably conflicting allegiances. The results provide an insight into this ‘balancing act’. Religious norms and the view that the early fetus has a moral right to life count against providing abortion; the interests of the pregnant woman count for it. This kind of experienced conflict has been described in other developing countries, such as Kenya [20]. Similarly, a study from South Africa indicated that abortion providers formed their views on abortion in light of personal, moral and religious factors [21].

Seen this way, it is natural that the professionals weigh the interests differently and have different positions. One would expect that the (presumably large) portion of healthcare professionals who have held on to a traditional ethical condemnation of abortion avoid employment in healthcare institutions where they would be expected to perform or assist with abortions. These are not represented in the study. However, it is interesting to see that also among those who have chosen to work with abortion, many have a troubled, ambiguous and/or unresolved attitude towards abortion. This corroborates McLean et al.’s finding [14], and also Yang et al.’s interviews with Taiwanese nurses [22].

Among the participants, a common view was that life in a biological sense starts at conception. This was so even though they all went on to defend abortion in at least some situations as a matter of ethics, professional duty and law. This in itself does of course not imply any contradiction. However, it was also found that some participants contradicted themselves throughout the interview, perhaps indicating that these were topics that they had not necessarily thought much about, at least not in these terms. One interpretation of the contradictions and hesitation seen with several of the participants is that not all the participants have given the issue that much thought. Although abortion is controversial in Ethiopia and hotly debated, starting to work in abortion services apparently has not necessarily ‘forced’ some of the participants to critically reflect on ethical dilemmas involved. Apparently, the ‘balancing act’ required to reconcile the conflicting norms and duties was one that each practitioner would have to perform on their own. No participant mentioned any communal deliberation about the dilemmas they faced. In our view, healthcare education ought explicitly to address the dilemmas of conflicting norms, values and duties in issues connected with abortion, to aid future practitioners in developing their own views. Furthermore, a safe forum for moral deliberation and discussion might be of help to some professionals.

One group appears to have experienced genuine conflicts of conscience. Here, some have also felt the need to conceal the true nature of their work from neighbors and acquaintances, as was also found among some of McLean et al.’s participants [14]. A significant group had attempted to reconcile religious norms and values with their work. In this group there appeared to be two argumentative strategies. One part of the group admitted that the abortion practices they were engaged in actually did contradict religious norms, but that the arguments *for* the practices were stronger. Here, several point to their own experiences of how lack of access to safe and legal abortion have caused suffering, complications and death for women. Apparently, such experiences were powerful lessons for several practitioners, leading them towards greater acceptance of legal abortion. Another strategy involved juxtaposing the religious prohibition on abortion with norms and duties of rescuing and helping, and pointing out that also the latter are valid religious considerations. This strategy, then, involves interpreting the demands of the religion, using its inherent ethical resources to show that it can also justify a practice of providing abortion to those with a significant need for it. This argumentative strategy might be thought to parallel how several Western Christian denominations nuanced their teaching on the ethics of abortion throughout the 20th and early 21st centuries [23].

In general, participants employed in the private sector displayed less anguish and discomfort with abortion as a religious-ethical dilemma than did their colleagues from public hospitals. If there is indeed a substantial difference between the groups, one explanation could perhaps be that those in the private sector have abortion as a larger part of their job, and thus their choice to work with abortion has been more of an active choice.

### Different views on the biological and moral status of the fetus and the ethical and legal permissibility of abortion

The same conflation of issues which, arguably, is prevalent also in Western discussions of the abortion issue leads to some apparent or genuine contradictions. Perhaps in future research the questions should be framed differently. It might be that it would have been helpful for the participants in the interview situation if from the start the interviews had been structured around four explicit questions; i.e. i) biology, ii) moral status, iii) *moral* acceptability of abortion, and iv) *legal* acceptability of abortion. As for the question of biology, most participants stated that the fetus was in fact human and that biological life started at conception. With regard to the second question on what moral status the fetus has at different stages of development, we find both the view that moral status (and a corresponding right to life) starts at conception, and the view that moral status comes later, either gradually throughout the pregnancy, at viability, or at birth.

With the third question about whether and when abortion is morally acceptable, we see that the interests of the pregnant woman come to the fore [24], and several participants, from the private clinics, asserted that the interests of the woman should outweigh considerations for the fetus and its moral status. Finally, the fourth question about when the law ought to allow abortion shows that it is possible to support a liberal abortion law yet maintain that most abortions are in fact morally problematic. In our study, most participants did not distinguish explicitly between the domains of law (question iv) and ethics (questions ii and iii).

An additional question is whether the experienced moral dilemmas and anguish lead the professionals to treat patients in a different manner than they would have if they had felt no moral qualms connected to abortion, as suggested by several studies [14, 16] This topic will be addressed in a future article.

### Limitations

Limitations inherent to the study merit discussion. While findings may be relevant to other similar settings, they cannot be generalized because of the purposive nature of the sample. All participants worked in Addis Ababa which may have introduced selection bias; recruiting participants from other parts of the country might have added depth to the findings. The first author is Ethiopian, trained in physiology and in Orthodox Christian theology, and is well conversant with such religious practices, which may have biased the way questions were phrased and translated. To minimize this effect, interview questions were developed in close collaboration with the co-authors; supervisor debriefing sessions were also held during the analysis phase; and an Ethiopian researcher independently translated all the transcribed interviews to assess quality and accuracy.

## Conclusion

Though circumscribed in scope, this study contributes to the research on abortion in low-income countries. It documents some of the complexities in reconciling value tensions (or paradox) perceived and expressed by participants, and ways Ethiopian healthcare professionals involved in abortion services try to balance their different allegiances and concerns. The study indicates that several experience conflicts of conscience. Such insights might inform guidelines and healthcare ethics education.

## Data Availability

In order to protect participants’ anonymity, the data (transcripts) will not be shared.

## Abbreviations

GYN/OBS: Gynecology/obstetrics
NGO: Non-governmental organization
STC: Systematic text condensation
